# Distinct Mechanism of Audiovisual Integration With Informative and Uninformative Sound in a Visual Detection Task: A DCM Study

**DOI:** 10.3389/fncom.2019.00059

**Published:** 2019-08-29

**Authors:** Qi Li, Yang Xi, Mengchao Zhang, Lin Liu, Xiaoyu Tang

**Affiliations:** ^1^School of Computer Science and Technology, Changchun University of Science and Technology, Changchun, China; ^2^School of Computer Science, Northeast Electric Power University, Jilin, China; ^3^Department of Radiology, China-Japan Union Hospital of Jilin University, Changchun, China; ^4^School of Psychology, Liaoning Normal University, Dalian, China

**Keywords:** audiovisual integration, fMRI, informativity of sound, DCM, effective connectivity

## Abstract

Previous studies have shown that task-irrelevant auditory information can provide temporal clues for the detection of visual targets and improve visual perception; such sounds are called informative sounds. The neural mechanism of the integration of informative sound and visual stimulus has been investigated extensively, using behavioral measurement or neuroimaging methods such as functional magnetic resonance imaging (fMRI) and event-related potential (ERP), but the dynamic processes of audiovisual integration cannot be characterized formally in terms of directed neuronal coupling. The present study adopts dynamic causal modeling (DCM) of fMRI data to identify changes in effective connectivity in the hierarchical brain networks that underwrite audiovisual integration and memory. This allows us to characterize context-sensitive changes in neuronal coupling and show how visual processing is contextualized by the processing of informative and uninformative sounds. Our results show that audiovisual integration with informative and uninformative sounds conforms to different optimal models in the two conditions, indicating distinct neural mechanisms of audiovisual integration. The findings also reveal that a sound is uninformative owing to low-level automatic audiovisual integration and informative owing to integration in high-level cognitive processes.

## Introduction

The brain receives multisensory information from the surrounding environment every moment. This information, in close coordination and with coincident temporal patterns, will be combined to enhance perceptual clarity and reduce ambiguity about the real world (Calvert et al., 2001; Ernst and Bulthoff, 2004; Bischoff et al., 2007). For example, it has been demonstrated that combining multiple senses can speed up reaction times (RTs) (Schröger and Widmann, 1998; Forster et al., 2002), facilitate learning (Seitz et al., 2006), and change the qualitative sensory experience (Jousmaki and Hari, 1998; Shams et al., 2000). Many studies have investigated the cross-modal effects of visual perception when a visual stimulus is accompanied by a task-irrelevant auditory signal, and the results have shown that concurrent auditory information cannot be completely ignored, and it might enhance visual perception (Frassinetti et al., 2002a; Lippert et al., 2007; Wu et al., 2009).

Lippert et al. (2007) investigated the effects of task-irrelevant sound on visual detection using a behavioral measurement. Their results showed that the sound reduced the time uncertainty of the visual display and significantly improved the performance of visual detection, in which case, the sound was informative. When a simultaneous visual cue (a rectangle) was presented around the visual target, the visual cue indicated the timing of the visual target to the participants and rendered the sound information redundant; thus, the behavioral benefit of the sound disappeared, in which case, the sound was uninformative (Lippert et al., 2007). Recently, Li et al. (2017) adopted a similar experimental design and further analyzed the neural mechanism of cross-modal integration for visual stimulus with an informative and an uninformative sound using functional magnetic resonance imaging (fMRI). Their results showed that the bilateral superior temporal gyrus/middle temporal gyrus (STG/MTG) was involved in the integration of the visual stimulus with both informative and uninformative sounds, and the right lateral occipital complex (LOC) was activated more strongly by the audiovisual stimulus compared to visual input alone in the informative sound condition. The authors suggested that the right LOC was modulated by the temporal relationship within the audiovisual stimulus and formed an audiovisual memory, resulting in high-level multisensory integration—which is a process related to experience, properties of the stimulus, and task content—and enhancement of behavioral responses (Li et al., 2017). Other studies proposed that low-level automatic integrations formed the basis of the informativity of sound (Stein et al., 1996; Vroomen and de Gelder, 2000; Frassinetti et al., 2002a, b; Teder-Salejarvi et al., 2005). For example, a simultaneous tone improved the detection of a dimly flashed light (McDonald et al., 2000; Vroomen and de Gelder, 2000; Frassinetti et al., 2002a, b; Teder-Salejarvi et al., 2005), enhanced the discriminability of briefly flashed visual patterns (Vroomen and de Gelder, 2000), and increased the perceived luminance of light (Stein et al., 1996).

Since a concurrent sound could facilitate visual detection in an informative sound condition but not in an uninformative sound condition, we anticipated that the neural mechanism of cross-modal integration for auditory and visual stimuli would be distinct in these two conditions. Moreover, cross-modal integration may involve low-level automatic or high-level cognitive processing, or it may occur by virtue of several mechanisms (McDonald et al., 2000; Frassinetti et al., 2002b; Wu et al., 2009; Li et al., 2017). However, behavioral measurement can only provide an indirect way to explain information processing in the brain, and fMRI results can present the activation areas to obtain the neural responses indirectly. However, it cannot show the dynamic information interaction among cortical areas, which is a more efficient way to reveal the neural mechanism of audiovisual integration and reflect information processing at low- or high-level stages. Fortunately, the interaction of multisensory information that underlies audiovisual processing can be characterized in terms of changes in directed effective connectivity among activated areas (Daunizeau et al., 2011). In the context of neuroimaging, inferences about the condition-specific change in connectivity can be made using Dynamic Causal Models (DCM) (Friston et al., 2003).

Here, we adopted fMRI data from the experiment of Li et al. (2017) about the informativity of sound, to construct effective brain networks for audiovisual integration and memory using DCM (Li et al., 2017). We then used a Bayesian model comparison to identify the optimal models for the informative and uninformative sound conditions. In particular, we were able to identify where changes in directed coupling within the auditory and visual hierarchies were sensitive to combined audiovisual input, as compared to visual input alone. By analyzing the optimal models under informative and uninformative sound conditions, we were also able to compare the selected models to assess the hierarchical level of changes in effective connectivity induced by audiovisual integration. These results will provide insight into the neural mechanism of the informativity of sound and cross-modal integration and might be useful for the detection and prevention of neurological diseases (Wu et al., 2012; Yu et al., 2016; Lei et al., 2018).

## Materials and Methods

### Participants

Fifteen volunteers (seven women, aged 22–25 years; mean age: 23.6 years) participated in the study. All participants had normal or corrected-to-normal vision and hearing. After receiving a full explanation of the experiment and potential risks, the participants provided written informed consent according to a protocol approved by the ethics committee of the Changchun University of Science and Technology (Li et al., 2017). All methods in our research were performed in accordance with the approved guidelines.

### Procedure

**FIGURE 1:**
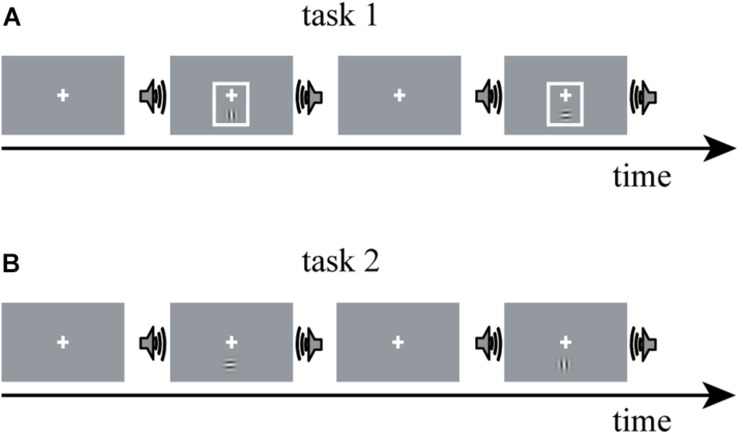
The experimental paradigm design of Li et al. (2017). The experiment comprises two tasks: V stimulus is a horizontal or vertical Gabor grating; AV stimulus is a Gabor grating and a concurrent task-irrelevant sinusoidal tone. **(A)** In task 1, a visual cue—a thin light gray frame—was presented simultaneously with the V stimulus; **(B)** task 2 was similar to task 1, except that the visual cue was not presented. V stimulus, visual stimulus; AV stimulus, audiovisual stimulus.

The fMRI data for constructing DCM models in the present study were obtained from a study by Li et al. (2017). Thus, here, we only summarize the experimental procedure for better understanding. A detailed description of the experimental design and parameter setting can be found in Li et al. (2017). Horizontal and vertical Gabor gratings were used as a visual (V) stimulus; and half of the V stimuli were accompanied by a task-irrelevant sinusoidal tone, termed as the audiovisual (AV) stimulus. The experiment contained two tasks, in each of which V or AV stimuli with horizontal and vertical Gabor gratings each accounted for 50%. In task 1, a visual cue—a thin light gray frame—was presented simultaneously with the V stimulus (Figure 1). The additional visual cue indicated the timing of the visual target and rendered the sound uninformative. Task 2 was similar to task 1, except that the visual cue was not presented (Figure 1). An event-related fMRI design was adopted. The participants were required to attend to V stimuli while ignoring the sound and were instructed to press the left button of a computer mouse when the presented V stimulus was a horizontal Gabor grating and press the right button when the presented V stimulus was a vertical Gabor grating.

### Processing of fMRI Data

Imaging data were analyzed using the SPM8 software package (Wellcome Department of Cognitive Neurology, London, United Kingdom) running under Matlab 2012a (MathWorks, Inc., Natick, MA, United States). Six scans at the beginning of the measurement were removed automatically from the data set. Functional data were time-corrected reference to middle slice and motion-corrected with realignment to the first image volume in the series, then normalized to standard anatomical space as defined by the Montreal Neurological Institute atlas and smoothed using an 8.0-mm full-width half-maximum Gaussian kernel.

**FIGURE 2:**
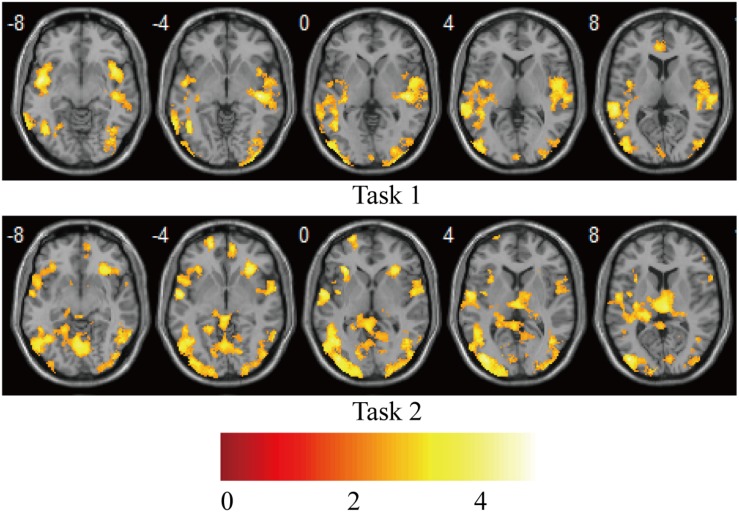
Brain activations on group-level for “AV stimulus” contrast in tasks 1 and 2. *P*_*uncorr*_ < 0.0005; extent threshold >30 voxels. The color bar indicates the *Z* scores.

Statistical analysis was carried out at the first level using a general linear regression model. The blood oxygen level–dependent response was modeled as the neural activity convolved with a canonical hemodynamic response function, which yielded two regressors for the “V stimulus” and “AV stimulus” in each task. After model estimation, individual contrast images for each of the 15 participants were generated for the “V stimulus” and “AV stimulus.” The contrast images from the first-level analyses were then used for the second-level group statistics. One-sample *t*-tests were used to construct statistical parametric maps at the group level for “AV stimulus” contrast in tasks 1 and 2 (Figure 2).

### Dynamic Causal Modeling

Dynamic causal modeling is a method of creating effective brain networks that can investigate audiovisual integrations in terms of changes in effective connectivity among the regions of interest (ROIs) (Gibson and Maunsell, 1997). DCM treats the brain as a dynamic input-state-output system (Werner and Noppeney, 2010; Cardin et al., 2011). There are three types of parameters in a DCM: (1) input parameters, which describe how much brain regions respond to experimental stimuli; (2) intrinsic parameters, which characterize effective connectivity among regions; and (3) modulatory parameters, which characterize changes in effective connectivity caused by experimental manipulation. DCM can reveal the network of effective connectivity and their context- or condition-specific changes (Cardin et al., 2011).

Here, we identified the ROIs for each participant based on the activation areas for “AV stimulus” contrast. The STG/MTG is the multisensory area modulated by the properties of AV stimulus, especially temporal factors (Calvert et al., 2000; Beauchamp et al., 2004; Stevenson et al., 2007), and it is involved in the audiovisual integration in both the informative and uninformative sound conditions (Li et al., 2017). The primary auditory cortex (PAC) and primary visual cortex (PVC) are generally considered to be sensory-specific regions that deal with unisensory visual and auditory information. However, some studies have suggested that even the primary cortical areas receive inputs from other unisensory areas or multisensory associative areas, exhibiting some abilities of multisensory processing (Schroeder and Foxe, 2005; Ghazanfar and Schroeder, 2006; Musacchia and Schroeder, 2009). In order to investigate the differences in processing of audiovisual integration in informative and uninformative sound conditions, the STG/MTG, PAC, and PVC were selected as ROIs to construct the DCM models. In addition, the right LOC was reported to be involved in the formation of cross-modal associations and the congruent AV memory (Gibson and Maunsell, 1997; Murray et al., 2004; Naghavi et al., 2011), which may relate to the informativity of sound (Li et al., 2017). Thus, the right visual area 3 (V3) in task 2 (informative sound condition), as a part of the right LOC, was selected to explore the audiovisual memory particular in the informative sound condition along with the other three ROIs (STG/MTG, PAC, and PVC). The ROIs were centered on the coordinates with prominent activation of the areas we selected (STG/MTG, PAC, and PVC in tasks 1 and 2; right V3 in task 2), using spheres 6 mm in diameter. Table 1 shows detailed information for the ROIs selected in the present study.

**TABLE 1:** Detailed information about the ROIs.

**Tasks**	**Regions**	**Coordinate left (x, y, z)**	***z*-value**	**Coordinate right (x, y, z)**	***z*-value**	**Brodmann area**
Task1	PAC	−54, −20, 10	4.66	56, −24, 12	10.41	41/42
	PVC	−12, −102, 8	6.51	16, −100, 4	5.37	17/18
	STG/MTG	−49, −17, 8	4.44	60, −26, 14	8.34	21/22
Task 2	PAC	−64, −28, 16	4.73	66, −14, 12	6.04	41/42
	PVC	−37, −86, −8	2.81	34, −82, −8	3.24	17/18
	STG/MTG	−50, −54, 10	7.35	42, −4, −10	11.72	21/22
	V3			37, −69, −19	5.18	19

**P*_*uncorr*_ < 0.0005; extent threshold >30 voxels. PAC, primary auditory cortex; PVC, primary visual cortex; STG/MTG, superior temporal gyrus/middle temporal gyrus; V3, visual area 3.*

We investigated dynamic audiovisual interaction by constructing DCM in two parts: effective networks of audiovisual integration and audiovisual memory. First, we constructed effective networks of audiovisual integration using the three ROIs (STG/MTG, PAC, and PVC for tasks 1 and 2). These networks were used to compare the interaction of visual stimuli with informative and uninformative sounds in a traditional audiovisual integration network (Werner and Noppeney, 2010). Second, we constructed the effective network of audiovisual memory using four ROIs (STG/MTG, PAC, PVC, and V3 for task 2), focusing on the interaction of the visual input and the informative sound in right V3.

#### The Effective Network of Audiovisual Integration

For each participant, four DCMs (Friston et al., 2003) were constructed for the left and right hemispheres, respectively. Each DCM included ipsilateral three regions of the PAC, PVC, and STG/MTG. The three ROIs were bidirectionally connected (Werner and Noppeney, 2010), with visual stimuli entering as extrinsic inputs to the PVC and AV stimulus as a modulatory factor.

**FIGURE 3:**
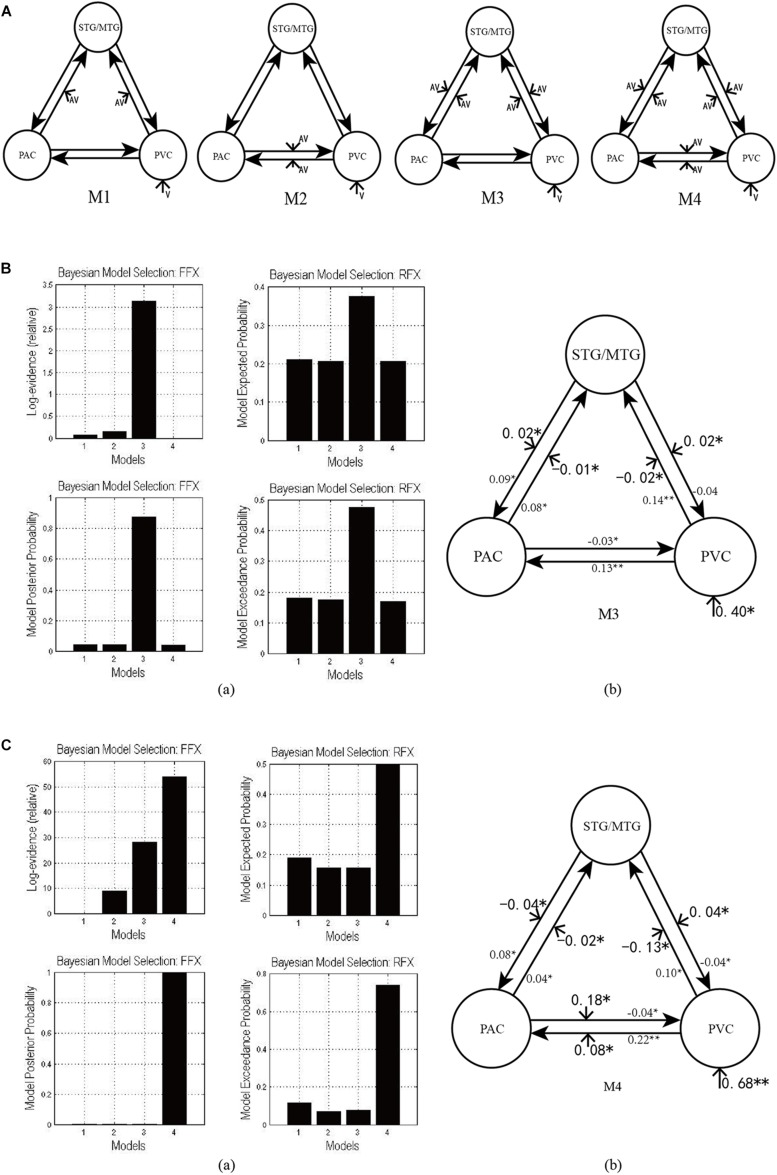
**(A)** Four candidate DCMs for the effective network of audiovisual integration represent AV stimulus modulating different intrinsic connectivity. **(B)** The results of model comparison and parameter estimation for M1–M4 for task 1, in which the sound is uninformative. **(a)** Model comparison for FFX and RFX, both indicating that M3 is the optimal model. **(b)** Parameter estimation of the connectivity in optimal model M3. **(C)** The results of model comparison and parameter estimation of M1–M4 for task 2, in which the sound is informative. **(a)** Model comparison for FFX and RFX, both indicating that M4 is the optimal model. **(b)** Parameter estimation of the connectivity in optimal model M4. ^∗∗^*p* < 0.01, ^∗^*p* < 0.05.

Figure 3A shows the four potential DCM candidates, differing with respect to condition-specific change in connectivity that was modulated by AV stimulus. Holding intrinsic and extrinsic structure constant, the DCMs manipulated this change in four ways. M1 is a typical feedforward model. It models the properties of higher-order multisensory area that receive converging inputs from lower-order unisensory areas, and it measures the influence of convergence on the responses in the multisensory area (Lim et al., 2011). M2 is a model with bidirectionally condition-specific change in connectivity between unisensory areas. One of the most intriguing properties of the cortex is that multisensory integration has already been exhibited in the primary unisensory areas (Calvert et al., 1999; Foxe et al., 2000). To investigate this aspect, M2 is proposed, a model specifically devoted to analyzing cross-modal interaction in unisensory areas (Magosso et al., 2012), including the bidirectional condition-specific change in direct connectivity between visual and auditory neurons. M3 is a feedforward-feedback model, incorporating feedforward to a multisensory area and feedback from there to the unisensory areas (Ursino et al., 2009). M4 is a model in which AV stimulus modulates all the intrinsic condition-specific changes in connectivity. The model consists of two lower-order unisensory areas and one higher-order multisensory area, reciprocally connected by feedforward and feedback projections. The lower-order unisensory areas also communicate via direct transverse condition-specific change in connectivity between them (Magosso et al., 2010; Hoshino, 2011).

These DCMs enable us to arbitrate between two main hypotheses currently advanced in multisensory research for audiovisual integration in the informative and uninformative sound conditions. One is that cross-modal integration in lower-order unisensory areas may be mediated via recurrent loops from higher-order convergence areas (Noesselt et al., 2007). The other is that cross-modal integration may be mediated via transverse condition-specific change in connectivity between lower-order unisensory areas (Ursino et al., 2017).

#### The Effective Network of Audiovisual Memory

For each participant, we constructed three DCMs using the data from task 2, including the PAC, PVC, STG/MTG, and V3 in the right hemisphere. Their structures were based on the theory that the output tracts of the occipital lobe mainly consists of two bundles, that is, the descending longitudinal nerve bundles reaching the temporal lobe along the ventral pathway, and the ascending longitudinal nerve bundles selecting a pathway closer to the back of the parietal lobe, most of which reach the posterior part of the parietal lobe. The dorsal pathway starts at V1 and runs to the middle temporal regions (MT, also called V5) through V2 and V3, and then reaches the inferior parietal lobule, which means that there is a condition-specific change in connectivity between V1 and V3 for visual information transmission. Some studies have revealed that the right V3 (as a part of the right LOC) is related to the formation of congruent AV memory (Gibson and Maunsell, 1997; Murray et al., 2004; Naghavi et al., 2011). When the sound and visual targets were presented simultaneously, there was a consistent temporal relationship between them. Li et al. (2017) considered that the activation of the right V3 was due to a short-term memory being generated by temporally congruent audiovisual stimulus. Therefore, since there was a condition-specific change in connectivity between V1 and V3, we inferred that there would also be condition-specific change in connectivity between the PAC and V3 for auditory information inputting into V3 and forming AV memory.

**FIGURE 4:**
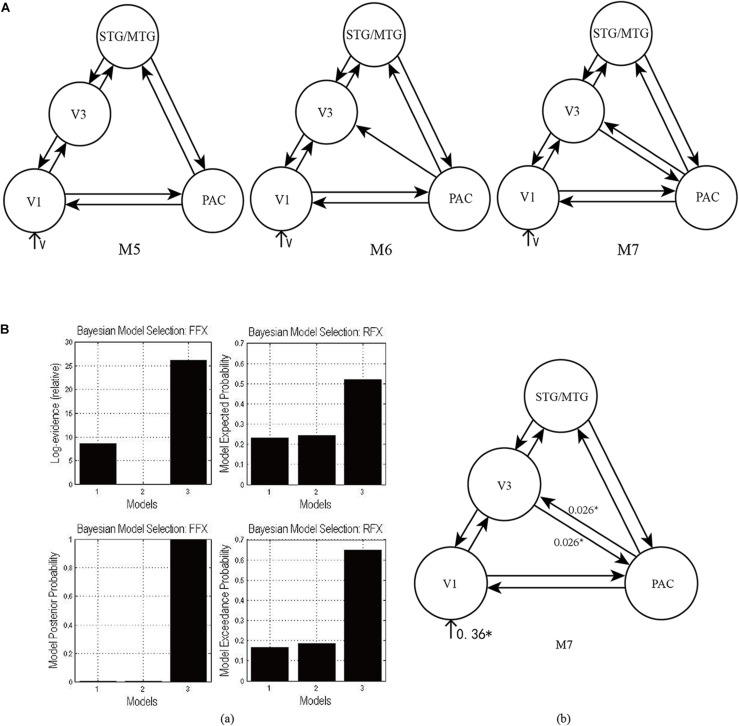
**(A)** Three candidate DCMs for the effective network of audiovisual memory represented no connectivity, a unidirectional connectivity, and bidirectional connectivity between the right V3 and PAC, respectively. **(B)** The results of model comparison and parameter estimation for M5–M7 for task 2, in which the sound is informative. **(a)** Model comparison for FFX and RFX, both showing that M7 is the optimal model. **(b)** Parameter estimation of the connectivity in optimal model M7. ^∗^*p* < 0.05.

As shown in Figure 4A, according to the possible direction of audiovisual interaction between the PAC and V3, three DCMs—M5, M6, and M7—were constructed. The difference between the three DCMs was whether there was connectivity between the PAC and V3, and whether the connectivity was unidirectional or bidirectional. Through model comparison, we can obtain the optimal model to verify one of the three hypotheses, that is, whether there is audiovisual interaction between the PAC and V3, and if so, whether the interaction is unidirectional or bidirectional.

### Bayesian Model Comparison

To determine the most likely DCM given the observed data from all participants, we implemented a fixed-effects (FFX) and a random-effects (RFX) group analysis (Friston et al., 2003). We used a posterior probability of 0.85 to identify differences for which there was strong evidence. Model comparison for M1–M4 would identify the optimal model of audiovisual integration in the informative and uninformative sound conditions. Model comparison for M5–M7 focused on the interaction between unisensory auditory and visual areas, and the formation of audiovisual congruency memory. For the optimal model, the participant-specific intrinsic, modulatory, and extrinsic effects were entered into one-sample *t*-tests using SPSS 22 (IBM Corp., Armonk, NY, United States). Statistical analyses with 95% confidence interval of connectivity parameters were used to quantify how simultaneous AV stimuli modulated connectivity. This allowed us to summarize consistent findings from the participant-specific DCMs using classical statistics. The classical analysis was used to provide quantitative estimates of the effect sizes, having accounted for variability among participants.

## Results

We constructed effective networks of audiovisual integration and audiovisual memory, respectively. The effective networks of audiovisual integration for the data in tasks 1 and 2 were focused on comparing the dynamic interaction of visual stimulus with informative and uninformative sounds. For the effective networks of audiovisual memory, we used only the data in task 2 to explore the dynamic interaction of visual stimulus and informative sound in the right LOC, which is involved in the formation of congruent audiovisual memory.

### The Effective Network of Audiovisual Integration

As shown in Figure 3Ba, for task 1, in which the sound was uninformative, both FFX and RFX analyses revealed M3 as optimal of the four models tested (FFX analysis: posterior probability for M3 was 0.88; RFX analysis: exceedance probability for M3 was 0.43). The intrinsic and extrinsic connectivity structure and the changes of connectivity strength due to AV stimulus for the optimal model M3 appear in Figure 3Bb. Not surprisingly, visual stimulus induced a positive activation in the PVC via extrinsic connectivity of visual input to the PVC with a value of 0.40 (*p* < 0.05). The AV stimulus modulated the feedforward from the PAC to the STG/MTG and from the PVC to the STG/MTG, and the feedback from the STG/MTG to the PAC and from the STG/MTG to the PVC. In particular, the modulation of AV stimulus to the information input from the PAC to the STG/MTG (−0.01, *p* < 0.05) and from the PVC to the STG/MTG (−0.02, *p* < 0.05) are negative, indicating that simultaneous audiovisual stimuli inhibited the information interaction from the unisensory areas to the multisensory area, and reduced the sensitivity of multisensory area (STG/MTG) to unisensory auditory and visual inputs (Werner and Noppeney, 2010). Moreover, AV stimulus enhanced the strength of condition-specific change in connectivity from the STG/MTG to the PAC (0.02, *p* < 0.05) and from the STG/MTG to the PVC (0.02, *p* < 0.05), improved the effect of multisensory area on unisensory areas, and further activated the PAC and PVC.

As shown in Figure 3Ca, for task 2, in which the sound was informative, both FFX and RFX analyses revealed M4 as the optimal of the four models tested (FFX analysis: posterior probability for M4 was nearly 1; RFX analysis: exceedance probability for M4 was 0.43). Figure 3Cb shows the structure of optimal model M4 and the connectivity parameters of M4. The visual stimulus induced a positive activation in the PVC via the extrinsic connectivity of visual input to the PVC with the value 0.68 (*p* < 0.01). Unlike the optimal model M3 in the uninformative sound condition, in M4, AV stimulus modulated not only the connectivity between the PAC and STG/MTG and the PVC and STG/MTG but also the bidirectional condition-specific change in the connectivity between the PAC and PVC. In particular, the modulation of AV stimulus to the information input from the PAC to the STG/MTG (−0.02, *p* < 0.05) and from the PVC to the STG/MTG (−0.13, *p* < 0.05) are negative, indicating that simultaneous audiovisual stimuli inhibited the information interaction from the unisensory visual and auditory areas to the multisensory area, and reduced the sensitivity of the STG/MTG to unisensory auditory and visual inputs (Werner and Noppeney, 2010). Moreover, AV stimulus enhanced the strength of bidirectional connectivity between the PAC and PVC with values 0.18 (*p* < 0.05) and 0.08 (*p* < 0.05), which meant that AV stimulus enhanced the audiovisual integration between unisensory areas.

### The Effective Network of Audiovisual Memory

As shown in Figure 4Ba, for task 2, both FFX and RFX analyses revealed that of the three models, M7 was optimal (FFX analysis: posterior probability was nearly 1; RFX analysis: exceedance probability was 0.92). Model comparison and parameters estimation revealed that bidirectional intrinsic connectivity between the PAC and V3 (as a part of the LOC) occurred in the right hemisphere with the value 0.026 (*p* < 0.05) (Figure 4Bb). The optimal model M7 proved that the audiovisual interaction between PAC and V3 indeed exists and provided a basis for the proposal (Li et al., 2017) that auditory and visual information formed a short-term memory related to their temporal relationship in the right V3.

### The Validity of the Optimal Model

**FIGURE 5:**
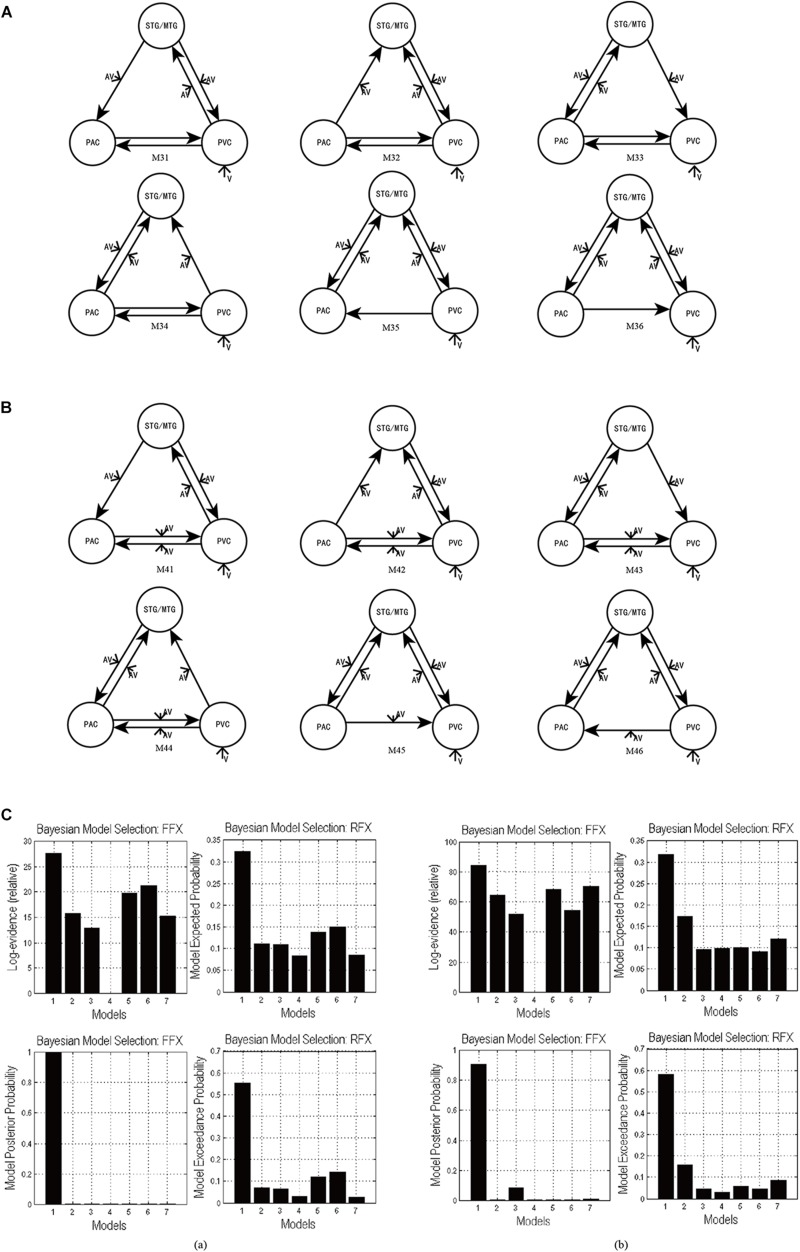
**(A)** The six DCMs M31–M36 constructed by removing one intrinsic link at a time from the optimal model M3 in task 1. **(B)** The six DCMs M41–M46 constructed by removing one intrinsic link DCMs from the optimal model M4 in task 2. **(C)** The result of model comparison for FFX and RFX. **(a)** The results of model comparison in task 1. The bar height represents the probability of each tested model (from left to right: M3, M31, M32, M33, M34, M35, and M36). **(b)** The results of model comparison in task 2. The bar height represents the probability of each tested model (from left to right: M4, M41, M42, M43, M44, M45, and M46).

In the candidate models M1–M4, all ROIs are bidirectionally connected with each other. Based on models M1–M4, we obtained the optimal model with bidirectional intrinsic connectivity for tasks 1 and 2. To test the validity of the intrinsic connectivity in each of the optimal models, we further constructed six models by removing one intrinsic link at a time from the optimal models. These six models were then compared with the optimal model that contains all the intrinsic links between ROIs. Specifically, for task 1, we further constructed six models, M31–M36, by removing one intrinsic connectivity at a time from the optimal model M3 (Figure 5A). Similarly, for task 2, we also constructed six models, M41–M46, by removing one intrinsic connectivity at a time from the optimal model M4 (Figure 5B).

Using FFX and RFX analyses for the Bayesian model comparison, the results showed that M3 was still the most optimal of the seven models (M3, M31–M36) tested for the uninformative sound condition (Figure 5Ca), and M4 is still the most optimal of the seven models (M4, M41–M46) tested for the informative sound condition (Figure 5Cb). These results reveal that intrinsic connectivity in optimal models M3 and M4 is valid and that the structure of the optimal models is reasonable; further, these optimal models can explain the audiovisual integration pattern in the uninformative and informative sound conditions.

## Discussion

We have taken a slightly unusual approach to Bayesian model comparison in establishing the distinct functional structures that underlie the multisensory integration of visual stimuli with informative and uninformative sound. Usually, one would compare models in which the change in directed connectivity—under combined AV input—depended upon the informative versus uninformative nature of auditory input; in other words, we would have compared models of the data from both experiments. However, in our case, the two tasks precluded this straightforward application of Bayesian model comparison, and we elected to conduct the comparison separately for the informative and uninformative conditions. This means that we cannot provide direct evidence that informative auditory input has differential effects; however, we can assert that under informative auditory conditions, there is clear evidence for changes of a hierarchical sort (at multiple levels of the multimodal hierarchy) that was not found during uninformative integration. Meanwhile, we verified the validity of the architectures of the optimal models with regard to the intrinsic connectivity, and the results showed that our optimal models (M3 for task 1 and M4 for task 2) could effectively reflect the pattern of audiovisual integration in the uninformative and informative sound conditions. Our findings revealed distinct neural mechanism of audiovisual integration in informative and uninformative sound conditions, and also shed light on dysfunctional connectivity in neurodegenerative diseases and neuropsychiatric disorders (Yu et al., 2016; Lei et al., 2018).

### Audiovisual Integration With Uninformative Sound

For the uninformative sound condition in task 1, the optimal model M3 showed that AV stimuli modulated the bottom-up connectivity from the PAC to the STG/MTG and from the PVC to the STG/MTG, and the top-down connectivity from the STG/MTG to the PAC and PVC (Figure 3B). A previous ERP study suggested that audiovisual information integrates in early-stage processing through an indirect pathway, in which feedforward auditory input reaches the areas of multisensory convergence and is transmitted via feedback to unisensory visual areas (Ursino et al., 2015). Similarly, visual input also reaches areas of multisensory convergence, and it is transmitted via feedback to unisensory auditory areas. Moreover, a behavioral study reported that the reaction times (RTs) of visual target detection did not significantly improve when the sound was uninformative, but the hit rates (HRs) had significantly increased (Li et al., 2017). It is generally considered that RTs are sensitive to late-stage cognitive manipulation, whereas HRs are affected by early-stage perceptual factors and reflect the degree of freedom and sensitivity of the participants to target detection (Mordkoff and Egeth, 1993; van Ede et al., 2012). In the study of Li et al. (2017) on which our present study is based, uninformative sounds only facilitated HRs, not RTs, of visual target detection. Altogether, we suggest that the modulation of AV stimulus to the bidirectional condition-specific change in connectivity between unisensory and multisensory area in the present study reflects a low-level automatic integration in early-stage processes. An fMRI study by Noesselt et al. (2010) showed that uninformative sound and lower-intensity visual targets integrate in primary visual and auditory cortices, and also in subcortical structures of lateral geniculate bodies (LGBs) and medial geniculate bodies (MGBs). Moreover, their analyses found enhanced effective connectivity between left LGB and ipsilateral primary visual cortex (PVC), as well as left MGB and ipsilateral Heschl’s gyrus (HG). These results reveal that the integration of uninformative sound and visual stimuli may involve low-level information processing from thalamus structures (LGB/MGB) to cortical areas (PVC and HG), which supports our conclusion that concurrent task-irrelevant sound is uninformative owing to low-level automatic audiovisual integration.

### Audiovisual Integration With Informative Sound

For the informative sound condition in task 2, model comparison showed that visual stimulus and informative sound integrate through three pathways in M4. (1) Direct interaction through the bidirectional condition-specific change in the connectivity between the PAC and PVC results in audiovisual integration (Figure 3C). (2) Auditory and visual stimuli input to the STG/MTG from the PAC and PVC, respectively. The integration occurs in the multisensory area, and the feedback is projected to the unisensory areas (Figure 3C). (3) Auditory stimulus interacts with visual stimulus in the right V3 region through bidirectional pathways between the PAC and right V3 (Figure 4B).

Until a few decades ago, most neuroscience texts assumed that individual senses are first processed separately in unisensory areas to extract typical information for each of them, and only subsequently combined at later processing stages in multisensory association areas. This theory, often named “unisensory before multisensory” (Ursino et al., 2015), is still valid in part. However, recent data, especially concerning the primary cortices, challenge this traditional view, showing that even the primary cortical areas receive inputs from other unisensory areas, multisensory associative areas or thalamus structures (Campi et al., 2010; Starke et al., 2017), and they exhibit some abilities of multisensory processing (Schroeder and Foxe, 2005; Ghazanfar and Schroeder, 2006; Musacchia and Schroeder, 2009). Studies with fMRI and neurophysiology have accumulated a lot of evidence demonstrating the existence of multisensory integration between the lower-order unisensory regions. For example, studies referring to multisensory integration in the auditory cortex have shown that both visual and somatosensory information can influence auditory neurons (Schroeder and Foxe, 2005; Musacchia and Schroeder, 2009; King and Walker, 2012). Bizley and King (2009) observed that the receptive field in the auditory cortex of animals could be affected by a visual input; spatially coincident visual and auditory stimuli often result in a more sharply-tuned auditory receptive field. Starke et al. (2017) found the early interaction of visual and auditory signals in the low-level auditory cortex, potentially mediated by crosstalk at the level of the thalamus. Several neuroanatomical studies report direct anatomical connections between subdivisions of the thalamus and auditory and other cortical areas (Cappe et al., 2009; Campi et al., 2010). Tyll et al. (2011) demonstrated that sensory-specific thalamic nuclei might integrate different sensory stimuli and influence cortical multisensory processing by means of thalamocortical feedforward connections. Regarding the visual cortex, early studies by Morrell (1972) reported that part of a cat’s visual neurons could be affected by an auditory input. Anatomical tracing studies in monkeys have demonstrated audiovisual convergence in the primary visual cortex (Poremba et al., 2003; Ghazanfar and Schroeder, 2006).

In the present study, the optimal model in the informative sound condition, M4, indicated that the audiovisual information integrates through not only the unisensory–multisensory pathways but also the unisensory-unisensory pathways, which did not exist in the uninformative sound condition (see model M3). Anatomical and physiological studies have reported that anatomical synaptic connectivity between the primary auditory and visual cortices exists, providing physiological basis for direct audiovisual interaction between unisensory areas (Ursino et al., 2017). Furthermore, synapses between the primary auditory and visual cortices are plastic, and the strength of the synapses indicates the experience of establishing a relationship for audiovisual stimulus (Falchier et al., 2002; Rockland and Ojima, 2003; Driver and Noesselt, 2008; Ursino et al., 2017). Interestingly, Lippert et al. (2007) declared that the participants’ “knowledge” about the relationship between a sound and a visual target is a crucial factor affecting the informativity of sound, because they found that the sound facilitates the detection of the visual target only when the participants are clearly aware of a consistent temporal association between concurrent sound and visual stimulus. In addition, some studies have shown that the activation of neurons in the unisensory areas, caused by information integration between them, determined the activation to the audiovisual stimulus in the multisensory area (Ursino et al., 2017). In other words, the cross-modal integration of information between unisensory areas determined, to a large extent, the results of cross-modal information processing in the multisensory area. Therefore, we believe that the synaptic connections between the primary auditory and visual cortices reflect the participants’ knowledge—or experience-dependent sensory learning—that the audiovisual information is temporally congruent, which is essential for the cross-modal facilitation of visual target detection.

Considering the condition-specific change in connectivity between the multisensory area and unisensory areas in M4, the bottom-up connectivity from the PAC to the STG/MTG and from the PVC to the STG/MTG represented the early-stage processes; however, it was hard to say whether the top-down connectivity from the STG/MTG to the PAC and PVC reflected automatic or cognitive processes in the informative sound condition. We speculate that the auditory and visual information integrate reciprocally via the recurrent network of multisensory–unisensory (PAC–STG/MTG, PVC–STG/MTG) and unisensory–unisensory (PAC-PVC) until the brain identifies a coherent percept. However, further studies are needed to confirm and elucidate these details.

Finally, the optimal model M7 revealed that there are bidirectional condition-specific changes in connectivity between the PAC and V3 in the right hemisphere. The visual stimulus and informative sound converge into the right V3, which is considered to be a region that generates audiovisual congruency traces and forms a short-term memory based on their temporal relationship (Li et al., 2017); the formation of memory involves high-level cognitive processes, modulated by attention. Therefore, the optimal model M7 supports the conclusion mentioned above, that audiovisual integration in the informative sound condition involves high-level cognitive processes.

### The Neural Mechanism of Informativity of Sound

Recently, more and more studies using both fMRI data analysis and computer simulations revealed that the dynamic functional connectivity and hierarchical brain networks are the vital basis for flexible functional integration in the brain (Lei et al., 2018; Song et al., 2019; Wang et al., 2019). In our study, the optimal model of audiovisual integration changes from M3 when the sound is uninformative to M4 when the sound is informative, showing the different connectivity patterns, which reveals distinct neural mechanism of audiovisual integration in these two conditions. When the sound is uninformative, the unisensory areas process the unisensory visual and auditory information separately, while the multisensory area is responsible for processing audiovisual integration, which involves automatic low-level processing. In this condition, unisensory areas do not process multisensory information, and the activation of the unisensory areas only depends on the extrinsic unisensory stimulus and feedback from the multisensory area, instead of the influence from the other unisensory area.

One particular cognitive task may require several brain regions to participate; meanwhile, one particular brain region may be responsible for several different cognitive abilities (Tagliazucchi et al., 2016; Song et al., 2019). In this sense, each brain region processes many different patterns of connections with other regions and dynamically updates them for different tasks (Betzel et al., 2015; Song et al., 2019). Consistent with this view, when the sound is informative, the audiovisual information changes the integration not only between the unisensory and multisensory areas, but also between the unisensory areas. An effective temporal association is established between concurrent sound and visual target stimuli (Lippert et al., 2007; Li et al., 2017), enhancing the strength of the synaptic link between unisensory areas and then promoting the directed transversal effective connectivity. In this condition, the unisensory areas exhibited multisensory behaviors. Audiovisual information integrates through the two pathways mentioned above until identifying a coherent percept, involving both low-level automatic and high-level cognitive processes, of which the latter one plays a crucial role in the informativity of sound. Furthermore, the audiovisual information also changes the coupling between the right V3 and PAC to form congruent AV memory when the sound is informative, involving high-level cognitive processes.

## Conclusion

In conclusion, our present DCM study showed the distinct neural mechanism of audiovisual integration with informative and uninformative sounds in a simple visual detection task. Our results revealed that the capability of unisensory auditory and visual areas for information processing changes, according to the informativity of sound, and that the audiovisual integration between unisensory areas is crucial for the informativity of sound. This study also supported the contention that informative sound is attributed to high-level cognitive processes, while uninformative sound is owing to low-level automatic processes.

## Data Availability

The raw data supporting the conclusions of this manuscript will be made available by the authors, without undue reservation, to any qualified researcher.

## Author Contributions

QL and LL designed the study. QL, YX, and XT analyzed the data. YX, QL, and MZ wrote the manuscript.

## Conflict of Interest Statement

The authors declare that the research was conducted in the absence of any commercial or financial relationships that could be construed as a potential conflict of interest.
